# Action verbs are processed differently in metaphorical and literal sentences depending on the semantic match of visual primes

**DOI:** 10.3389/fnhum.2014.00982

**Published:** 2014-12-04

**Authors:** Melissa Troyer, Lauren B. Curley, Luke E. Miller, Ayse P. Saygin, Benjamin K. Bergen

**Affiliations:** Department of Cognitive Science, University of CaliforniaSan Diego, La Jolla, CA, USA

**Keywords:** sentence processing, verbal semantics, point-light walkers, biological motion, metaphor

## Abstract

Language comprehension requires rapid and flexible access to information stored in long-term memory, likely influenced by activation of rich world knowledge and by brain systems that support the processing of sensorimotor content. We hypothesized that while literal language about biological motion might rely on neurocognitive representations of biological motion specific to the details of the actions described, metaphors rely on more generic representations of motion. In a priming and self-paced reading paradigm, participants saw video clips or images of (a) an intact point-light walker or (b) a scrambled control and read sentences containing literal or metaphoric uses of biological motion verbs either closely or distantly related to the depicted action (walking). We predicted that reading times for literal and metaphorical sentences would show differential sensitivity to the match between the verb and the visual prime. In Experiment 1, we observed interactions between the prime type (walker or scrambled video) and the verb type (close or distant match) for both literal and metaphorical sentences, but with strikingly different patterns. We found no difference in the verb region of literal sentences for Close-Match verbs after walker or scrambled motion primes, but Distant-Match verbs were read more quickly following walker primes. For metaphorical sentences, the results were roughly reversed, with Distant-Match verbs being read more slowly following a walker compared to scrambled motion. In Experiment 2, we observed a similar pattern following still image primes, though critical interactions emerged later in the sentence. We interpret these findings as evidence for shared recruitment of cognitive and neural mechanisms for processing visual and verbal biological motion information. Metaphoric language using biological motion verbs may recruit neurocognitive mechanisms similar to those used in processing literal language but be represented in a less-specific way.

## Introduction

A central question in the cognitive neuroscience of language is how meaning is represented and accessed in the brain during language comprehension and production. It has been well established that language and perception interact in the brain and in behavior. For instance, even after the presentation of a single word, semantically related words are processed more quickly (i.e., primed), possibly due to spreading activation of features shared between the two words (Anderson, 1983), and semantic priming occurs across mixed input and target modalities, such that pictures can also prime related words, and vice versa (Sperber et al., 1979). In the domain of action perception, a great deal of research supports the notion that visually perceiving actions and processing language about actions rely on overlapping representations in the mind and brain (Barsalou, 1999; Glenberg and Kaschak, 2002; Bergen et al., 2007). Here, we set out to explore the relationship between processing language about biological motion and visually perceiving it. To this end, we asked how a visual prime depicting biological motion would affect reading times of literal and metaphorical language containing action verbs with either a close or more distant semantic relationship to the prime.

Literal meaning is dependent not only on information stored in semantic memory but also on the physical context, including representations that are grounded in perception and action (Barsalou, 1999). Glenberg and Kaschak (2002) found that when people processed linguistic information about motion directed toward the self (e.g., *Close the drawer*), compared to away from the self (e.g., *Open the drawer*), they were faster to respond using a button in that direction (that is, closer to the self, compared to farther away). A subsequent study investigating this *action compatibility effect* (ACE) further probed its timing and found that when participants did not know which response button they would use prior to reading such sentences, compatibility effects were no longer present (Borreggine and Kaschak, 2006). These findings minimally suggest that processing language about actions can rely upon representations overlapping with those used to process the physical actions themselves but that the timecourse of such activations is sensitive to context.

Other studies have shown that literal language about objects and space can both facilitate (e.g., Stanfield and Zwaan, 2001) and inhibit (e.g., Richardson et al., 2003; Bergen et al., 2007, 2010) similar behavioral responses, largely dependent on the timing between primes and the response measure. Stanfield and Zwaan (2001) had participants read sentences about objects which implied an object's orientation (either horizontal, e.g., *John put the pencil in the drawer*, or vertical, e.g., *John put the pencil in the cup*). Next, participants were shown a picture of an object in either a horizontal or vertical orientation and were asked whether it had been mentioned in the preceding sentence. They observed that participants responded more quickly when the object matched the implied orientation of the object mentioned in the sentences.

Bergen et al. (2007) investigated the extent to which visual imagery is used in understanding both literal and metaphorical language and found interference-type effects. After reading a short sentence, participants categorized a shape presented in either the upper or lower part of the screen. When the sentence contained concrete verbs or nouns which were semantically associated with the concepts “up” (e.g., *The cork rocketed*) vs. “down” (e.g., *The glass fell*), participants were systematically slower to make a decision about an object presented in the associated part of the screen. These findings support the hypothesis that participants use visual imagery to process literal language about space. Taken together with other behavioral results suggesting that people use mental imagery or simulations when processing information about which effector, or body part, is being used in an action (Bergen et al., 2010), the shape of an object described (Stanfield and Zwaan, 2001), and the axis along which motion occurs (Richardson et al., 2003), these findings suggest people use partially overlapping representations for processing information about physical action and space and linguistic content about action and space.

Functional imaging studies have demonstrated support for this conclusion through comparisons of brain regions involved in sensory perception (e.g., tactile, visual) and motor function with the brain regions involved in processing language whose meaning may be derived using these modalities (Just et al., 2004; Boulenger et al., 2009; Moody and Gennari, 2009; Saygin et al., 2010; Willems et al., 2010). In the domain of motor output, Moody and Gennari (2009) found that a region in premotor cortex was sensitive to the degree of real-world effort required for the action described by a verb in a particular context (e.g., pushing a piano requires more effort than pushing a chair). Interestingly, the anterior inferior frontal gyrus, a region thought to be involved in semantic processing more generally (Demb et al., 1995; Kuperberg et al., 2008) (but not in motor processing, specifically), was also sensitive to the degree of effort. Furthermore, action words read on their own (e.g., *kick, lick*, etc.) have been shown to preferentially activate motor regions corresponding to the particular effector (Hauk et al., 2004). Using transcranial magnetic stimulation (TMS) to target specific areas of the motor strip, Pulvermüller et al. (2005) also observed selective interference for effector-specific regions in the motor strip for words related to those effectors. These findings suggest a role for the motor system in more general processing of linguistic content about action.

As for perceptual systems, Just et al. (2004) showed that, compared to low imageability sentences, highly imageable language (e.g., sentences like *The number eight when rotated 90 degrees looks like a pair of spectacles*) modulated activity in the intraparietal sulcus, a brain region thought to play a role in visual attention (Wojciuluk and Kanwisher, 1999) and visual working memory (Todd and Marois, 2004). Higher visual areas such as V5/MT+ have also been implicated in processing language about visual motion (Saygin et al., 2010), though others have found this region either to be unaffected by processing high-motion verbs compared to low-motion (Bedny et al., 2008) or implicated only in a minority of subjects for motion compared to static verbs (Humphreys et al., 2013).

This work converges to suggest that motor production and visual perception of motion are candidates for neurocognitive processes which may contribute to comprehending language about motion. It is now well-established that visually processing others' actions relies on neural mechanisms overlapping with those used for language processing (Rizzolatti et al., 2001; Arbib, 2005). In the present study, we focused in particular on *biological* motion, which refers to the characteristic movement patterns of animate entities as well as specific stimuli used in vision science to study its perception (see below; Johannson, 1973; Blake and Shiffrar, 2007). Producing and understanding biological motion are important functions for humans, and humans are sensitive to perceiving biological motion even when cues are relatively minimal, as with the point-light displays used in vision science (Johannson, 1973), which are animations showing only points placed over key joints of a moving person. Humans exhibit robust perception of biological motion even in degraded conditions (see Blake and Shiffrar, 2007, for a review).

Studies of biological motion perception using point-light walkers have most consistently implicated the posterior superior temporal sulcus (pSTS) as a key region (Grossman et al., 2000, 2005; Vaina et al., 2001; Beauchamp et al., 2003; Pelphrey et al., 2003; Puce and Perrett, 2003; Saygin, 2007; Vangeneugden et al., 2011; van Kemenade et al., 2012; Gilaie-Dotan et al., 2013). In addition, Kemmerer et al. (2008) found that the pSTS was specifically recruited more during silent reading of biological motion verbs similar to *run*, e.g., *jog, walk*, compared to other types of action verbs, implicating involvement of this region in both perception of visual biological motion and comprehension of language about such motion. Studies have also shown involvement of other regions in processing biological motion including parietal cortex, body and motion-sensitive visual areas in lateral temporal cortex (EBA, MT+), other areas in temporal and occipital cortex, and the cerebellum (Vaina et al., 2001; Grossman and Blake, 2002; Servos et al., 2002; Saygin et al., 2004b; Nelissen et al., 2005; Jastorff et al., 2010; Sokolov et al., 2012; Vangeneugden et al., 2014).

Importantly, regions that overlap with classical language areas in inferior frontal/ventral premotor cortex (PMC) have been linked to biological motion processing (Saygin et al., 2004b; Saygin, 2007; Pavlova, 2012; van Kemenade et al., 2012; Gilaie-Dotan et al., 2013). One interpretation of the role of frontal regions is that when people view point-light walkers, they recruit their own motor resources for performing the action, as suggested by proponents of embodied cognition and the “mirror neuron” system (Rizzolatti et al., 2001). That the premotor cortex, a brain region that supports the production of motor acts, is activated during perception of point-light walkers supports the notion that this process of motor simulation occurs even when the action is depicted via motion cues (Saygin et al., 2004b). The relationship between motor processing and biological motion perception is not purely correlational: disruption of processing in these regions due to brain injury or virtual lesions induced by TMS leads to deficits in biological motion perception (Saygin, 2007; van Kemenade et al., 2012). Furthermore, activation in these areas can be modulated in those who are experts at performing actions they are viewing, as in the case of professional dancers (Calvo-Merino et al., 2005; Cross et al., 2006). In the general population, individual differences in biological motion are predicted by individual levels of motor imagery (Miller and Saygin, 2013). Given the overlap between frontal regions involved in processing of visual biological motion on the one hand and language comprehension on the other, it is likely that language comprehension may benefit from recruitment of neurocognitive processes also involved in visual perception of motion.

There is therefore evidence that regions involved in both motor execution and visual biological motion perception can be recruited in understanding the semantics of literal language. Another active area of inquiry is the processing of metaphorical meaning. The range of linguistic use and experience extends far beyond the most literal uses of verbs like *throwing* and *walking* to uses in figurative and metaphorical contexts. Conceptual metaphor theory (Lakoff, 1993) proposes that metaphors are understood through a mapping from more concrete source domains (for instance, space) to more abstract target domains (for instance, time, as in, *Time is flying by*). This theory leads to predictions that the cognitive and neural representation of concrete verbs will be activated when they are used metaphorically due to the mapping from the source to target domains. For instance, in the sentence, *The movie was racing to its end*, the verb *racing* might activate brain regions involved in motor execution of running or racing.

Studies directly comparing literal and metaphorical language to investigate whether brain regions and cognitive processes involved in sensory and motor processing are recruited equally for both types of language have found mixed results. Bergen et al. (2007) observed null effects when investigating metaphorical language about space in their spatial interference paradigm. Unlike literal language, where interference effects were observed, metaphorical language related to the spatial concepts “up” (*The numbers rocketed*) and “down” (*The quantity fell*) did not lead to interference when participants made decisions about objects located in the upper or lower halves of a computer screen, respectively. These findings suggest no (or lesser) overlap for spatial representations and metaphorical language about space (compared to literal language about space).

However, other studies have provided evidence that non-literal uses of language about space and motion do recruit more general perceptuo-motor representations. For instance, Matlock et al. have argued that when participants read sentences involving fictive motion describing static events using motion verbs, they mentally simulate motion despite no implication of a physical change (Matlock, 2004; Richardson and Matlock, 2007). For instance, Matlock (2004) had participants read stories ending in a sentence containing a fictive motion verb (e.g., *The road runs through the valley*) and make judgments about whether the sentence made sense given the context. For fictive motion sentences (but not for literal sentences in control experiments, which did not contain fictive motion), the time to make the decision was dependent on properties of the sentence including the speed of travel, the distance traveled, and the ease or difficulty of terrain. These findings minimally suggest that even for language that doesn't imply true motion, individuals access motion-like properties when processing fictive motion verbs.

In a study of metaphorical uses of motion verbs, Wilson and Gibbs (2007) looked at how quickly phrases were read following real or imagined movements made by participants. Across two experiments, participants memorized a set of actions to be performed (Experiment 1) or imagined (Experiment 2) when they viewed particular symbols (e.g., the symbol “and” was paired with the action *push*). Then, they performed a task in which they viewed a symbol, either performed or imagined the action, and then read metaphorical language either related (e.g., *push the argument*) or not (e.g., *stamp out a fear*) to the action. For both performed and imagined actions, participants were faster to read phrases (as measured by the response time of a button press after reading the phrase) when the previous action was congruent with the verb. Wilson and Gibbs interpreted these findings as evidence that processing of metaphorical uses of action verbs relies on representations shared with executing their literal meanings physically. However, if individuals access lexical representations of verbs associated with performed or imagined actions (e.g., activating the word *push* while performing the action of pushing), this alone could be sufficient to prime reading of the phrase *push the argument* even if participants do not activate such representations during normal metaphorical language comprehension. An additional limitation of this study is that it provided no literal comparison (e.g., reading phrases liked *push the cart*, or even reading bare verbs like *push*). Such comparisons would be informative as to the extent to which such concepts (like *push*), when used metaphorically, recruit representations overlapping with those used in motor executions.

In the neuroimaging literature, evidence that metaphorical language recruits perceptuo-motor representations is also mixed. Some studies have found no evidence that processing metaphorical language about particular body parts, for instance, recruits brain regions involved in moving those body parts (Aziz-Zadeh et al., 2006; Raposo et al., 2009). One study using word-by-word reading of language (rather than whole-sentence reading) observed a relationship between both literal and idiomatic sentences involving different parts of the body and the corresponding somatotopic regions of the motor strip (Boulenger et al., 2009). For instance, sentences like *John grasped the object/idea* elicited stronger activity in the finger areas of motor strip while sentences like *Pablo kicked the ball/habit* elicited stronger activity in the foot areas of motor strip. However, this study involved a limited number of stimuli in each condition with many repetitions across the experiment, which limits the conclusions that can be drawn about typical sentence comprehension.

Other imaging studies have investigated fictive motion sentences, with mixed results. (such as *A crack was running along the wall* or *The pipe goes into the house*). One study found no difference in activations in visual motion perception regions for language describing fictive motion and actual motion (Wallentin et al., 2005). Saygin et al. (2010) individually located motion-sensitive areas as well as face-sensitive areas in each participant and compared brain activity in real motion, fictive motion, and static sentences which were presented as audiovisual movies of a person speaking the sentences. They observed a gradient pattern: actual motion sentences elicited the greatest amount of activity in visual motion perception areas, followed by fictive motion sentences and finally static sentences. This pattern of activity was not observed in face-sensitive areas, showing that the effect was indeed related to motion semantics. In another study, literal motion sentences also modulated activity in motion-sensitive areas for American Sign Language, suggesting that the concurrent recruitment of motion processing mechanisms during language processing does not abolish the effect (McCullough et al., 2012). These findings suggest that both literal and figurative language about motion recruits brain regions involved in visual motion perception, though to a lesser extent by the latter than the former.

Bergen (2012) suggests that mixed results in studies investigating recruitment of perceptuo-motor representations during metaphorical language comprehension may be due to the timecourse of processing. In many studies investigating metaphor (e.g., Aziz-Zadeh et al., 2006; Raposo et al., 2009), whole sentences are presented concurrently to the participant, a procedure which may not maintain the temporal precision necessary to observe effects. In other studies using either shorter phrases or word-by-word presentation of metaphorical language, effects have been observed both behaviorally (Wilson and Gibbs, 2007) and in neuroimaging data (Boulenger et al., 2009). Consider the following metaphorical sentence, an example from our materials: *The story was ambling toward its conclusion*. In natural spoken language, at the point of processing the word *ambling*, the listener may already have enough information (from having heard, first, *The story*) to know that the literal use of *ambling* is inappropriate; stories are abstract and have no legs. However, there are certain similarities that might be mapped out between *stories*, which cannot amble, and *people*, who can. A story takes place on a timeline, and people perceive a timeline through which life moves forward. There are therefore systematic relationships between the word *ambling* used metaphorically and *ambling* used literally. However, in the case of this metaphorical sentence, it is possible that the full metaphorical meaning might not be understood until the end of the sentence (after having heard *toward its conclusion*). That is to say, incrementally comprehending the meaning of such a metaphor might operate rather differently than incrementally comprehending the meaning of a similar literal sentence (e.g., *The teacher was ambling toward the school*). Further, while metaphorical language can recruit brain regions thought to underlie perceptuo-motor representations relevant for the literal use of the language, evidence suggests that these representations may not be as strongly activated for metaphorical language as for literal language (Saygin et al., 2010).

Here, we examined the extent to which processing visual biological motion affected the processing of motion verbs used in both metaphorical and literal contexts. We used short videos of point-light walkers (Johannson, 1973), which display representations of human biological motion, as primes. To determine the effect that recently viewing physical walking motion would have on processing closely-matching verbs (such as *ambling, walking*) compared to more distantly-matching verbs (such as *leaping, catapulting*), we also used a control motion condition with inverted, scrambled versions of the point-light walkers. A novel contribution of our approach is that the study fully crosses literal and metaphorical language (using identical verbs) with prime type (presence or absence of an action prime) and match-type of the verb use. Following other behavioral and neuroimaging research, we expected to see facilitation following the point-light walker during self-paced reading for literal sentences containing closely-matching verbs compared to those containing distantly-matching verbs. As for our metaphorical sentences, which used the same verbs as the literal sentences but in different contexts, we predicted that if processing visual biological motion is less involved (on average) when reading metaphorical language, we would observe a smaller difference in (or absence of) facilitation between closely-matching and distantly-matching verbs. Such findings would also indicate that processing metaphorical verbs might rely upon a less precise representation of the motion described by the verb.

## Experiment 1

### Methods

#### Norming studies

We conducted a norming study to prepare experimental materials. We created a set of 18 verbs intended to be descriptive of our point-light walker (this was called the Close-Match set) along with a set of 18 verbs intended to be much less descriptive of this action (this was called the Distant-Match set). For instance, *ambling* was included as a proposed Close-Match verb, and *catapulting* was included as a proposed Distant-Match verb. All Close- and Distant-Match verbs denoted biological motion and could describe an individual moving unidirectionally along a path. In addition, nine Control verbs were included in the norming study. These verbs also described biological motion, though of a very different nature from the type of motion depicting moving along a path (e.g., *shoving* and *sitting*). These verbs were included to provide variety in the set of verbs and also to act as a baseline comparison as biological motion verbs that should be the least likely to match the video.

Five volunteers rated all verbs for how closely they described the action being performed in a short video clip (the point-light walker; see below) on a scale of 1 (least similar to video clip) to 7 (most similar to video clip). For all five participants, verbs were presented in alphabetical order.

As predicted, Close-Match verbs were rated higher (*M* = 5.76, *SD* = 1.09) than Distant-Match verbs (*M* = 1.511, *SD* = 0.94), *t*_(4)_ = 10.34, *p* < 0.001. The nine highest-rated Close-Match verbs were chosen to be included as the verbs in the sentences used in the study. All of these verbs equaled or exceeded the mean of the total set (ratings for each provided in Table 1). Nine of the lowest 10 Distant-Match verbs were also included [one verb, *leapfrogging*, was discarded due to its low frequency, as determined by ratings from the MRC Psycholinguistic Database (Wilson, 1988)]. As for the control verbs (*M* = 1.64, *SD* = 1.05), these were not rated differently from the Distant-Match verb group, but were rated lower than the Close-Match verbs, *t*_(4)_ = 10.49, *p* < 0.001.

**Table 1:** **Ratings for Close-Match and Distant-Match verbs used in the experiment**.

**Verb**	**Category**	**Mean rating (Standard Deviation)**
Ambling	Close-Match	6.2 (0.84)
Meandering	Close-Match	5.8 (1.79)
Moseying	Close-Match	6.2 (0.84)
Plodding	Close-Match	5.2 (0.45)
Sauntering	Close-Match	5.0 (0.71)
Striding	Close-Match	6.2 (0.84)
Strolling	Close-Match	6.2 (0.84)
Walking	Close-Match	6.2 (1.10)
Wandering	Close-Match	4.8 (1.30)
Catapulting	Distant-Match	1.4 (0.55)
Hopping	Distant-Match	1.4 (0.55)
Leaping	Distant-Match	1.4 (0.89)
Skipping	Distant-Match	2.4 (1.52)
Springing	Distant-Match	2.6 (1.52)
Swimming	Distant-Match	1.0 (0.00)
Twirling	Distant-Match	1.2 (0.45)
Vaulting	Distant-Match	1.0 (0.00)
Whirling	Distant-Match	1.2 (0.45)

Using the 18 verbs from the first set of norms, two sentences were constructed for each verb, one literal and one metaphorical. Four volunteers rated these sentences for how natural they sounded on a scale from 1 (least natural) to 7 (most natural). In addition to the 36 experimental items (18 literal, 18 metaphorical), we included 12 Filler sentences to be used in the study as well as 12 additional implausible sentences which were only used for the norming study. Filler sentences had the same structure as experimental sentences, except that the verb was not a biological motion verb (e.g., *The teenager was learning in the classroom*.). Implausible sentences were included for variety and so that participants saw sentences specifically designed to elicit low ratings (e.g., *The bread was baking the chef*.). Crucially, both the literal (*M* = 5.96, *SD* = 1.40) and metaphorical (*M* = 4.74, *SD* = 1.43) sets were rated significantly higher than the implausible (*M* = 1.88, *SD* = 1.14) items, *t*_(3)_ = 5.16, *p* < 0.05 and *t*_(3)_ = 3.60, *p* < 0.05, respectively. A smaller (but reliable) difference was observed between the metaphorical and literal sets, *t*_(3)_ = 20.37, *p* < 0.001. This difference is important to consider in interpreting any overall differences in reading times for literal and metaphorical sentences observed in the reading time experiment. Numerically, filler sentences were rated the most natural (*M* = 6.56, *SD* = 0.85) and were rated higher than the metaphorical sentences [*t*_(3)_ = 4.96, *p* < 0.05], but not the Literal sentences.

#### Participants

Participants were 39 undergraduate students, ages 18–34 (*M* = 22, 27 female), at the University of California, San Diego. All participants reported that they were native English speakers and gave informed consent for the study, which was approved by the University of California, San Diego Institutional Review Board. Participants received partial course credit for participating in the experiment.

#### Materials

***Visual primes***. Stimuli were presented on a CRT screen using the Psychophysics Toolbox (Brainard, 1997; Pelli, 1997) for Matlab (refresh rate 60 Hz and screen resolution 1024 × 768 pixels).

**Figure 1:**
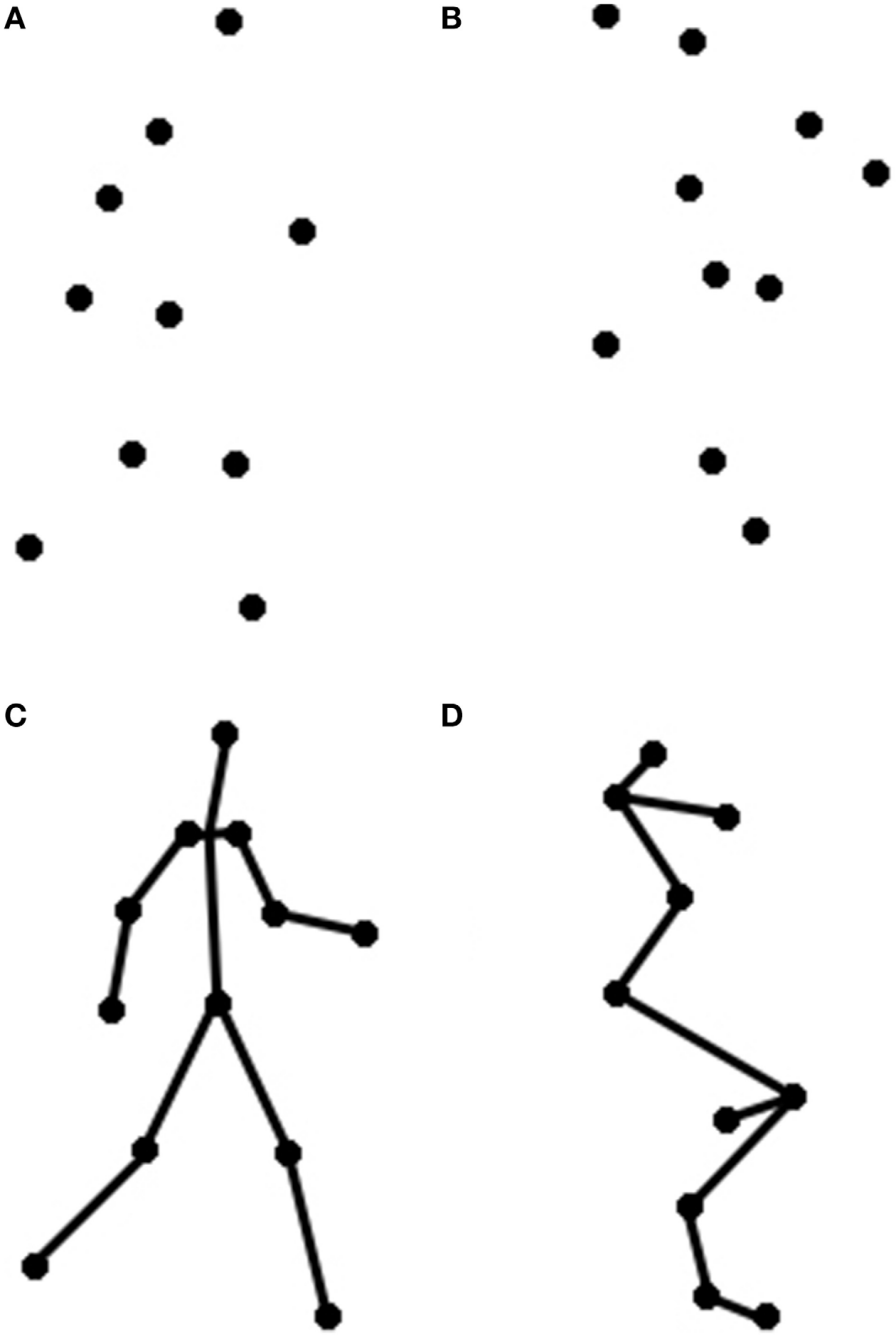
**These are depictions of the motion prime videos from Experiments 1 to 2, including both (A) Walker and (B) Scrambled primes, and of the static prime images from Experiment 2, including both (C) Walker, and (D) Scrambled primes**.

Two visual primes were used: an intact, coherent point light walker, and a scrambled point light display. The intact point-light walker was taken from the stimulus set reported in Ahlström et al. (1997). This walker was created by videotaping a human actor walking in place (on a treadmill) and recording the joint positions (e.g., elbow, wrist) of the whole body. The walker was composed of 10 black dots against a white background (see Figure 1). The height of the walker subtended approximately 5.6° of visual angle when viewed at a distance of approximately 91 cm. Given the bias of English speakers (who read left to right) to prefer and conceive of actions proceeding from left to right (Chatterjee et al., 1995, 1999; Christman and Pinger, 1997; Chatterjee, 2001), the walkers were always facing to the right.

To create a control prime, we inverted and spatially scrambled the individual points of the intact point-light walker so that the figure could no longer be seen as a person walking. For each trial, the starting position of each dot was pseudorandomly chosen within a rectangle subtending approximately 5.6° of visual angle viewed at a distance of 91 cm. in order to match the overall size and dimensions of the upright walker. This manipulation preserved important low-level features of the walker stimuli, including local motion information and point trajectories, while removing global motion information present in the intact walker. The final image appeared as a cluster of centralized dots following individual ellipsoidal paths, based on the same individual dot trajectories as the intact walker (see Figure 1). These stimuli have been used in previous studies of biological motion processing (Grossman and Blake, 2002; Saygin et al., 2004b; Miller and Saygin, 2013).

***Sentence materials***. Each participant read a total of 72 sentences: 36 experimental sentences as well as 36 Filler sentences. Twelve of the Filler sentences had been included in the second norming study, and 24 additional sentences were created so that the number of Filler sentences equaled the number of experimental sentences. Examples of sentences from each sentence condition (Literal, Metaphorical, and Filler) and Verb Match condition (Close-Match, Distant-Match) are presented in (1). Sentences were presented with center-screen self-paced reading, with regions indicated by slashes.

**Table:** 

(1)	Literal, Close-Match:	The teacher/was ambling/toward the school.
	Metaphorical, Close-Match:	The story/was ambling/toward its conclusion.
	Literal, Distant-Match:	The child/was hopping/to the swingset.
	Metaphorical, Distant-Match:	The melody/was hopping/to a high note.
	Filler:	The journalist/was scribbling/in his notebook.

Statistics for number of syllables, lexical frequency, concreteness, and imageability for each critical word were computed separately for each sentence type (literal, metaphorical). Critical words were (1) the subject noun (e.g., *teacher*), (2) the verb (e.g., *walking*), and the final noun of the prepositional phrase (e.g., *school*). Some nouns were compound, and in these cases, the nouns were not included in the analyses. Pairwise *t*-tests were then performed for each sentence type and critical word for close- vs. Distant-Match conditions. These statistics are provided in Supplementary Tables 1, 2. The only difference based on a lexical variable was for imageability, which was higher for Distant-Match nouns compared to close-match nouns for metaphorical sentences at the first critical word (the subject noun). This noun was rated as more highly imageable in the Distant-Match condition (*M* = 560) than in the close-match condition (*M* = 441), *t*_(6.935)_ = −3.4471, *p* < 0.05. All other tests revealed no differences between groups.

Each sentence was followed by a comprehension question, which the participant answered with *yes* or *no* using the keyboard. Half of the comprehension questions were correctly answered with *yes* and half with *no*.

#### Design and procedure

The experiment design was a 2 (Prime Type: Walker or Scrambled) × 2 (Verb Match: Close-Match or Distant-Match) × 2 (Sentence Type: Literal or Metaphorical). The materials were pseudo-randomized across three lists, such that each participant read each sentence exactly once. The type of visual prime (Walker or Scrambled) preceding the sentence varied across the three lists, such that an intact walker preceded two thirds of the experimental items and a scrambled walker preceded one third of the experimental items. The reasoning behind this choice in design was that the content of the scrambled walkers should be dissimilar to both Close- and Distant-Match verbs. However, the proportion of Filler sentences preceded by a scrambled visual prime was two thirds, with one third being preceded by a walker visual prime, so that across the experiment, all participants saw a total of 36 sentences presented by a walker prime and 36 sentences preceded by a scrambled prime.

On each trial of the experiment, a crosshair appeared in the center of the screen for 3 s (followed by an inter-stimulus interval (ISI) of 1200 ms). Participants then saw a visual prime (for approximately 1000 ms) followed by a display of three dashes in the center of the screen. This was the cue that they could begin reading the sentence for that trial at their own pace by pressing the space bar. A button press was always followed by a delay of 200 ms before the next region was presented. After participants read all three regions, they were presented with a comprehension question, which they answered with yes or no by pressing the “z” or “m” key, respectively. Participants were then given feedback (“Correct” or “Incorrect”).

#### Analysis

Statistical analyses used mixed-effects models incorporating both fixed and random effects and were performed using the lme4 package in the software program R. Fixed effects included Prime Type (Walker, Scrambled) and Verb Match (Close-Match, Distant-Match). Since each participant most likely displays some idiosyncratic behavior (for instance, some people may be faster readers than others), and since individual sentences may be more or less difficult to process (independent of their length), we treated both subject and item as random effects. In addition, phrase length (in number of characters) was also included as a fixed effect in all models. For most analyses, we consider Literal sentences separately from Metaphorical sentences, but in overall analyses, we also analyzed effects of Sentence Type (Literal, Metaphorical, or Filler). Finally, Markov Chain Monte Carlo (MCMC) sampling was used to estimate *p*-values for fixed effects using the pvals.fnc command in the lme4 package in R.

### Results

#### Reading times by sentence type

**Figure 2:**
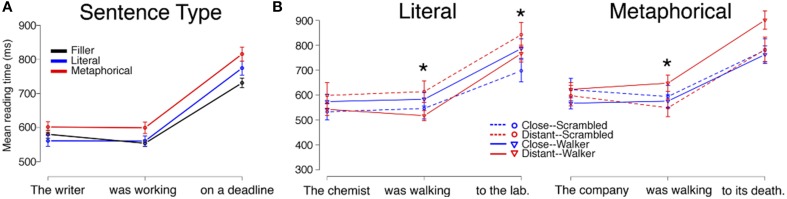
**Reading times for sentence regions from Experiment 1 are displayed by (A) Sentence Type and (B) by Verb Match and Prime Type for Literal and Metaphorical sentences**. Error bars represent standard errors of the mean. Interactions between Verb Match and Prime Type at the critical (second) region, which contained the verb, were observed for both literal and metaphorical sentences, but with different patterns. The ^*^ indicates a significant interaction of Match and Prime for that region (the reader is referred to the text for main effects and other statistics).

Mean reading times by region for each Sentence Type (Filler, Literal, Metaphorical) are displayed in Figure 2A. An analysis including Sentence Type (with Filler acting as the baseline in the model) and length of region as fixed effects and participant and item as random effects showed that there were differences based on Sentence Type in regions 2 and 3 (see Table 2). To examine these differences, we used follow-up mixed-effects linear regression models restricting comparisons to only two conditions at a time. Here, we found an increase in RTs for Metaphorical sentences in region 2 (the verb region) compared to both Literal sentences (β = 43.797, *SE* = 17.670, *t* = 2.479, *p* < 0.05) and Filler sentences (β = 56.195, *SE* = 15.28, *t* = 3.739, *p* < 0.001). Filler and Literal sentences did not differ in region 2. In region 3, Filler sentences led to faster reading times than both Metaphorical sentences (β = 102.316, *SE* = 22.568, *t* = 4.534, *p* < 0.001) and Literal sentences (β = 54.251, *SE* = 21.931, *t* = 2.474, *p* < 0.05) sentences, but only a marginal difference was observed between Literal and Metaphorical sentences at region 3 (β = 44.474, *SE* = 22.832, *t* = 1.948, *p* = 0.052).

**Table 2:** **Model estimates and statistics for analysis by Sentence Type from Experiment 1**.

**Region**	**Effect**	**Estimate**	**Std. Error**	***t*-Value**	***p*-Value**
Region 1	(Intercept)	512.300	54.964	9.321	0.0000
	SentenceTypeLit	−8.570	17.278	−0.496	0.6199
	SentenceTypeMet	31.866	17.169	1.856	0.0636
	Length (Region1)	5.762	3.253	1.771	0.0766
Region 2	(Intercept)	483.889	73.987	6.540	0.0000
	SentenceTypeLit	12.109	15.064	0.804	0.4216
	SentenceTypeMet	52.876	15.070	3.509	0.0005
	Length (Region2)	5.781	5.081	1.138	0.2553
Region 3	(Intercept)	741.302	70.011	10.588	0.0000
	SentenceTypeLit	54.751	21.432	2.543	0.0111
	SentenceTypeMet	99.497	22.411	4.440	0.0000
	Length (Region3)	−0.666	2.908	−0.229	0.8189

#### Literal sentences

Reading times for Literal sentence regions by condition are displayed in Figure 2B, and model estimates are provided in Table 3. For Literal sentences, there were no effects or interactions of prime or match type at region 1, the noun phrase.

**Table 3:** **Model estimates and statistics for the Literal sentences**.

**Region**	**Effect**	**Estimate**	**Std. Error**	***t*-Value**	***p*-Value**
Region 1	(Intercept)	486.888	138.153	3.524	0.0005
	Match type	68.631	53.770	1.276	0.2023
	Prime type	41.377	40.466	1.022	0.3069
	Length (Region 1)	3.902	11.289	0.346	0.7297
	Match × Prime	−91.609	57.453	−1.595	0.1113
Region 2	(Intercept)	454.478	183.970	2.470	0.0137
	Match type	67.379	46.184	1.459	0.1451
	Prime type	36.490	35.584	1.026	0.3055
	Length (Region 2)	7.257	14.208	0.511	0.6097
	Match × Prime	−129.880	50.522	−2.571	0.0104
Region 3	(Intercept)	607.871	181.862	3.342	0.0556
	Match type	138.852	72.416	1.917	0.0556
	Prime type	87.607	48.639	1.801	0.0721
	Length (Region 3)	5.192	9.889	0.525	0.5998
	Match × Prime	−163.964	69.054	−2.374	0.0179

At region 2, the verb phrase, we observed an interaction of Verb Match and Prime Type (β = 129.880, *SE* = 50.522, *t* = −2.571, *p* < 0.05), with no main effect of either Verb Match or Prime Type. To interpret this interaction, we then performed mixed-effects linear regression models on pairs of conditions. Only one significant difference was observed, with the Distant-Match sentences being read more quickly at region 2 following Walker primes compared to Scrambled primes (β = −93.18, *SE* = 30.45, *t* = −3.061, *p* < 0.001). This suggests facilitation for less closely matching verbs following the viewing of a walker.

Finally, at region 3, the final prepositional phrase, we observed marginal main effects of both Prime Type (β = 87.607, *SE* = 48.639, *t* = 1.801, *p* = 0.072) and Verb Match (β = 138.852, *SE* = 72.416, *t* = 1.917, *p* = 0.056), and a significant interaction of match and Prime Type (β = −163.964, *SE* = 69.054, *t* = −2.374, *p* < 0.05). Interpretation of this interaction is not straightforward. We again performed mixed-effects linear regression models on pairs of conditions to interpret this interaction. Although numerically, the largest difference was within the Scrambled prime conditions, there was no significant difference between the Scrambled/Close-Match and Scrambled/Distant-Match conditions (*p* = 0.14). However, there were marginal differences between prime types both within the Close-Match conditions (β = 87.850, *SE* = 51.468, *t* = 1.707, *p* = 0.089) and Distant-Match conditions (β = −78.296, *SE* = 43.735, *t* = −1.790, *p* = 0.074). In Literal sentences with Close-Match verbs, reading times trended toward being shorter after the Scrambled primes, possibly suggesting interference for verbs that closely matched the walker prime. In Literal sentences with Distant-Match verbs, however, reading times trended toward being faster after a Walker prime, suggesting that sentences with verbs which matched less closely to the Walker prime may have been easier to comprehend.

#### Metaphorical sentences

Reading times for Metaphorical sentences by region, Prime Type, and Verb Match type are displayed in Figure 2B, and model estimates and statistics are provided in Table 4. No significant effects or interactions of Prime Type or Verb Match were observed at region 1.

**Table 4:** **Model estimates and statistics for the Metaphorical sentences, Experiment 1**.

**Region**	**Effect**	**Estimate**	**Std. Error**	***t*-Value**	***p*-Value**
Region 1	(Intercept)	663.437	102.933	6.445	0.0000
	Match type	−33.977	55.000	−0.618	0.5369
	Prime type	−69.110	41.189	−1.678	0.0938
	Length (Region 1)	−2.691	7.919	−0.340	0.7341
	Match × Prime	89.942	58.127	1.547	0.1223
Region 2	(Intercept)	851.06	281.18	3.027	0.0026
	Match type	−59.69	62.34	−0.957	0.3387
	Prime type	−40.23	43.07	−0.934	0.3506
	Length (Region 2)	−19.38	21.92	−0.884	0.3769
	Match × Prime	132.30	61.00	2.169	0.0304
Region 3	(Intercept)	671.020	149.367	4.492	0.0000
	Match type	3.676	65.971	0.056	0.9556
	Prime type	−8.150	51.194	−0.159	0.8736
	Length (Region 3)	5.697	6.852	0.831	0.4060
	Match × Prime	128.441	71.540	1.795	0.0730

At region 2, an interaction of Prime Type and Verb Match was again observed (β = 132.30, *SE* = 61.00, *t* = 2.169, *p* < 0.05). To interpret this interaction, conditions were then compared by pair. Only one significant difference was observed, with the Distant-Match sentences being read more slowly at region 2 following Walker primes compared to the Scrambled primes (β = 99.14, *SE* = 45.77, *t* = 2.166, *p* < 0.05). This finding is the opposite of that observed in region 2 in the Literal condition, where the Distant-Match verb regions were read more quickly after Walker primes compared to the Scrambled primes.

At region 3, there was also a marginal interaction of Prime Type and Verb Match (β = 128.441, *SE* = 71.540, *t* = 1.795, *p* = 0.073). Subsequent paired tests showed that within only the Distant-Match conditions, region 3 was read more slowly for the Walker prime condition than in the Scrambled prime condition (β = 127.210, *SE* = 52.222, *t* = 2.436, *p* < 0.05). In addition, within the Walker prime conditions, the Distant-Match condition was read marginally more slowly than the Close-Match condition (β = 129.556, *SE* = 66.896, *t* = 1.937, *p* = 0.053).

#### Comprehension questions

Overall, comprehension question accuracy was high. Across all sentences (including fillers), mean accuracy was 96.70% (*SD* = 2.94%), with a range of 91.43–100%. There were no significant differences in accuracy by Sentence Type, Prime Type, or Verb Match type, nor any interactions of these variables. Mean accuracy for Filler sentences was 96.85% (*SD* = 3.42%); for Literal sentences, 96.42% (*SD* = 4.45%); and for Metaphorical sentences, 96.71% (*SD* = 4.62%).

### Discussion of experiment 1

Experiment 1 set out to ask whether comprehending visual depictions of biological motion and processing (a) literal and/or (b) metaphorical verbal material about biological motion recruit overlapping neurocognitive representations. Our findings suggest that at least part of these representations may be shared across visual and literal verbal modalities as well as across visual and metaphorical verbal modalities, though the precise representations used for processing verbal material may differ depending on the specific type of language being used (i.e., literal vs. metaphorical).

In Literal sentences, reading times were speeded at the verb region (region 2) following intact (compared to scrambled) walker primes for verbs which only distantly matched the action depicted in the prime. That is, a prime video showing a point-light display of a human figure walking led to faster reading times for the verb region of sentences containing verbs which had been rated as dissimilar to the action depicted in the video (e.g., verbs like *vaulting* or *catapulting*). Furthermore, at a subsequent region of the sentence (a final prepositional phrase), sentences containing closely-matching verbs (like *ambling* and *strolling*) were read more slowly following intact walker primes (compared to scrambled). Following intact walkers, Close-Match verb sentences were also read more slowly than Distant-Match verb sentences. These findings suggest that processing literal language about biological motion may rely on similar representations as the visual depiction of similar actions, such that there is interference in reading language shortly following the processing of a video with closely-matching visual content.

As for the Metaphorical sentences, reading times were *slowed* at the verb region (region 2) following intact (compared to scrambled) walker primes for verbs which only distantly matched the action depicted in the prime. At the final region, there were also slower reading times for sentences containing Distant-Match verbs following intact (compared to scrambled) walker primes, and there were longer reading times for Distant-Match verb conditions compared to Close-Match verb conditions following intact walker primes. These findings show a reversed pattern, compared to the Literal sentences: here, it appears that less-closely-matching verbal content shows interference. Tentatively, these findings imply broader (or less precise) representations for biological motion verbs being used metaphorically. That is, given that metaphorical use of biological motions verbs led to an increase in reading times for Distant-Match verbs following intact walker primes, it appears that these verbs led to interference in understanding language. This would be expected if metaphorical uses of biological motion verbs activate broader swaths of semantic content in memory. For example, although the verb *jogging* might evoke action-specific representations when it occurs literally, it might activate more general motion representations when used metaphorically, including representations that overlap with the visual depiction of a walker.

These findings converge to suggest that there is an overlap in the representations for biological motion content in both verbal material (i.e., comprehending sentences) and visual material (i.e., processing the video). However, whether the shared representations across visual and verbal modalities are driven primarily by motion *per se* or whether they rely on information about form (e.g., of a human walker) remains an open question. That is, which aspects of the videos are important for representations that may be accessed during language processing? It may be the case that seeing a still image which implies the presence of biological motion may be sufficient to activate representations from memory that may be useful for processing related language about biological motion. Indeed, neuropsychological and TMS studies have shown that injury in ventral premotor cortex leads to deficits in processing actions with or without motion (Saygin et al., 2004a; Pobric and Hamilton, 2006; Saygin, 2007). Furthermore, Grossman and Blake (2001) reported that imagined biological motion recruits brain regions implicated in processing biological motion such as the pSTS. Experiment 2 addresses this question by attempting to replicate Experiment 1 (which used visual biological motion primes) and by extending the paradigm to include a second group of participants who viewed still image primes of similar walkers in order to ask whether the priming effects seen in Experiment 1 were primarily due to viewing motion, or whether they could be induced by static content, where motion is only implied.

## Experiment 2

In Experiment 2, we sought to replicate and extend the finding from Experiment 1 that the processing of literal and metaphorical language about biological motion was affected by visual processing of biological motion in different ways. We also attempted to tease apart potential effects of visual form and visual motion on language processing. To this end, we randomly assigned half of our participants to view primes that were videos of point light displays (identical to those used in Experiment 1), and the other half saw primes that were static images created from similar displays of either randomly displayed dots or point-light walkers. We predicted that if the visual image of the form of a walker alone is enough to imply motion, then we should see nearly identical processing of language following still images compared to motion videos. If, however, visual form is not sufficient to activate neurocognitive representations of motion that may be accessed in language comprehension, then we may observe no priming effects for the still images (or smaller priming effects). We were also interested in the extent to which the still images might have a different effect on metaphorical and literal language, compared to the motion video clips. For instance, more-specific representations of biological motion might be accessed when processing literal biological motion language (such as *The teacher was ambling toward the school*.), but less-specific representations of biological motion might be accessed when processing metaphorical biological motion language (like *The story was ambling toward its conclusion*.). If this is the case, then we might see priming for only the metaphorical sentences following static primes, but not for literal sentences.

### Methods

#### Participants

Participants were 59 undergraduates, ages 18–32 (*M* = 21, 48 female) at the University of California, San Diego. Thirty participants saw motion video primes and 29 participants saw still image primes. All participants reported that they were native English speakers, and gave informed consent for the study, which was approved by the Institutional Review Board. Participants received partial course credit for participating in the experiment.

#### Materials

Stimuli were presented on a CRT screen using the Psychophysics Toolbox (Brainard, 1997; Pelli, 1997) for Matlab (refresh rate 60 Hz and screen resolution 1024 × 768 pixels).

Visual prime materials for the participants who saw motion primes (i.e., the Motion group) were identical to those used in Experiment 1. The still images used in this experiment (in the Static group) were taken from similar point-light walker displays (Vanrie and Verfaillie, 2004) and edited in Inkscape so that they subtended approximately the same visual angle as the videos (5.6° of visual angle viewed at a distance of approximately 91 cm). Scrambled images were also taken from screen captures of scrambled and inverted versions of the upright walkers. As before, images were presented on the screen with a small amount of jitter (up to 0.4° of visual angle) to prevent visual adaptation between trials.

All sentence materials were identical to those used in Experiment 1.

#### Design and procedure

Participants were placed into one of two groups: Motion (replicating Experiment 1) or Static. Within each group, the design was the same: 2 (Prime Type: Walker or Scrambled) × 2 (Verb Match: Close-Match or Distant-Match) × 2 (Sentence Type: Literal or Metaphorical). Other than different Motion and Static participants groups, the design and procedure were identical to those of Experiment 1.

#### Analysis

Motion and Static participant groups were analyzed separately. Analyses were otherwise identical to those used in Experiment 1.

### Results

#### Reading times by sentence type

**Figure 3:**
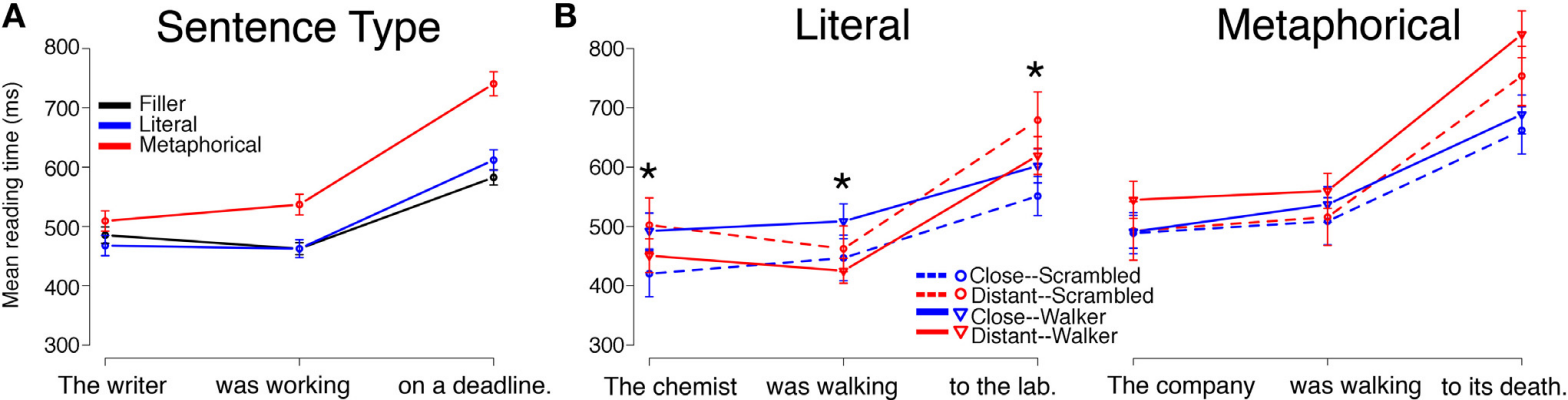
**Reading times for sentence regions from the Motion Group in Experiment 2 are displayed by (A) Sentence Type and (B) by Verb Match and Prime Type for Literal and Metaphorical sentences**. Error bars represent standard errors of the mean. Interactions between Verb Match and Prime Type at the critical (second) region, which contained the verb, were observed for Literal sentences whereas no significant main effects or interactions were observed in this region for Metaphorical sentences. The ^*^ indicates a significant interaction of Match and Prime for that region (the reader is referred to the text for main effects and other statistics).

**Figure 4:**
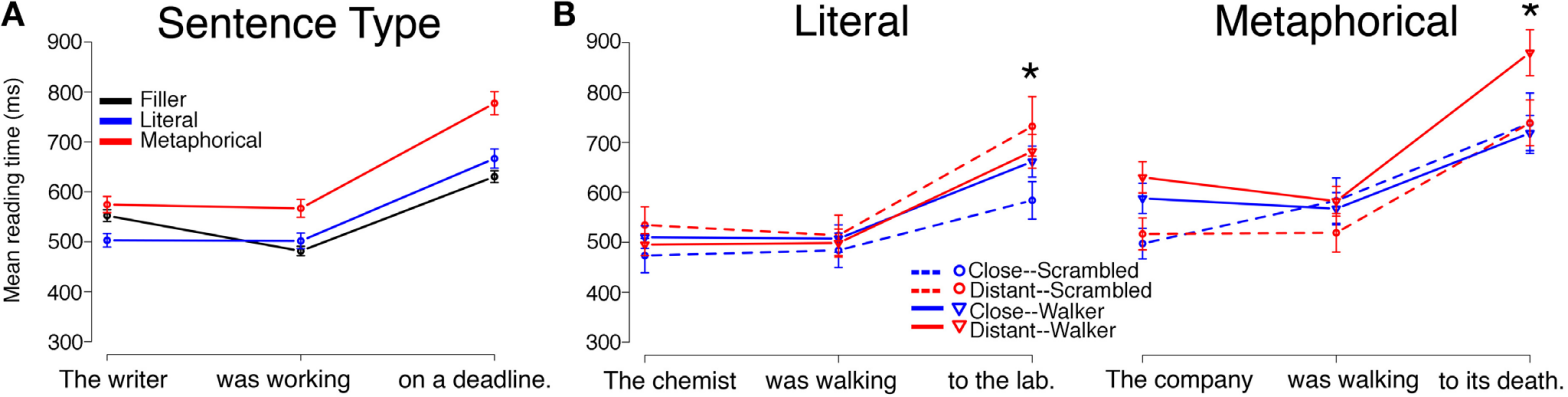
**Reading times for sentence regions from the Static Group in Experiment 2 are displayed by (A) Sentence Type and (B) by Verb Match and Prime Type for Literal and Metaphorical sentences**. Error bars represent standard errors of the mean. No significant main effects or interactions between Verb Match and Prime Type were observed at the critical (second) region for either the Literal or Metaphorical sentences. However, interactions for both sentence types emerged at region 3, with different patterns. The ^*^ indicates a significant interaction of Match and Prime for that region (the reader is referred to the text for main effects and other statistics).

Reading times by Sentence Type (Literal, Metaphorical, Filler), collapsed across Verb Type and Prime Type, followed a pattern similar to that of Experiment 1 for both the Motion group and for the Static group, with minor differences. Mean reading times by region for each Sentence Type (Filler, Literal, Metaphorical) are displayed in Figure 3A (Motion group) and Figure 4A (Static group).

For the Motion group, differences emerged at all three regions, with Metaphorical sentences being read slower than the other types of sentences at all three regions. Pair-wise comparisons using linear mixed-effects models revealed that at region 1, Metaphorical sentences were read more slowly than Literal sentences (β = 43.766, *SE* = 71.202, *t* = 6.329, *p* < 0.01) and read marginally slower than Filler sentences (β = 29.461, *SE* = 15.864, *t* = 1.857, *p* = 0.06). At region 2, Metaphorical sentences were read more slowly than both Literal sentences (β = 77.458, *SE* = 15.695, *t* = 4.935, *p* < 0.001) and Filler sentences (β = 73.177, *SE* = 13.703, *t* = 5.340, *p* < 0.001). This pattern persisted at region 3, with Metaphorical sentences being read more slowly than both Literal sentences (β = 127.910, *SE* = 19.328, *t* = 6.618, *p* < 0.001) and Filler sentences (β = 128.612, *SE* = 20.301, *t* = 6.335, *p* < 0.001). There were no differences between reading times for Literal sentences and Filler sentences in any of the regions.

For the Static group, differences emerged at all three regions. The metaphorical condition was again read the slowest across regions. At region 1, Metaphorical sentences were read more slowly than both Literal sentences (β = 71.734, *SE* = 16.012, *t* = 4.480, *p* < 0.001) and Filler sentences (β = 36.062, *SE* = 16.803, *t* = 2.146, *p* < 0.05). Literal sentences were also read marginally more slowly than Filler sentences at this region (β = −27.602, *SE* = 15.845, *t* = −1.742, *p* = 0.08). At region 2, Metaphorical sentences were read more slowly than both Literal sentences (β = 67.076, *SE* = 17.578, *t* = 3.816, *p* < 0.001) and Filler sentences (β = 99.378, *SE* = 14.713, *t* = 6.754, *p* < 0.001). However, Literal sentences were read more slowly than Filler sentences at region 2 (β = 33.198, *SE* 13.5, *t* = 2.459, *p* < 0.05). The pattern at region 3 was the same as at region 2. Metaphorical sentences were read more slowly than both Literal sentences (β = 112.684, *SE* = 22.922, *t* = 4.916, *p* < 0.005) and Filler sentences (β = 140.385, *SE* = 20.755, *t* = 6.764, *p* < 0.001). Literal sentences were read more slowly than Filler sentences at region 3 (β = 42.40, *SE* = 18.49, *t* = 2.281, *p* < 0.05).

#### Reading time differences for motion vs. static groups

To determine whether the Group Type (i.e., whether participants saw motion vs. static primes) had an effect on overall reading time, we analyzed reading times for each sentence region as a function of Sentence Type (Literal, Metaphorical, or Filler sentences) and Group (Motion, Static). As discussed in the previous section (Reading times by Sentence Type), significant differences emerged based on Sentence Type, but there were no main effects of Group Type or interactions of Group Type and Sentence Type (all *p*s > 0.10).

#### Literal sentences, motion group

Mean reading times for the Literal sentences for the Motion group are plotted in Figure 3B. The Literal sentences in the Motion group serve as a direct replication of the Literal sentences from Experiment 1. However, in contrast to Experiment 1, at region 1, there was a main effect of Verb Match (β = 90.941, *SE* = 44.879, *t* = 2.026, *p* < 0.05), a main effect of Prime Type (main effect of walker type), and an interaction of Verb Match and Prime Type (β = −122.107, *SE* = 43.225, *t* = −2.825, *p* < 0.01). These findings suggests that the Distant-Match verb sentences were read more slowly at region 1; that the Scrambled primes led to overall slower reading times at region 1; and that there was an interaction of the two factors. Given that Close- and Distant-Match sentences began with different words, even at region 1, it is possible that subtle differences in the noun phrases used in these conditions contributed to the differences observed in region 1.

As in Experiment 1, we observed an interaction between Prime Type and Verb Match at region 2, the verb region (β = −98.22, *SE* = 39.02, *t* = −2.517, *p* < 0.05). In addition, we also observed a main effect of Prime Type in this region (β = 61.65, *SE* = 27.47, *t* = 2.245, *p* < 0.05). The main effect of Prime Type suggests that overall, Literal sentences were read slower at the verb following Walker primes (compared to Scrambled primes). However, the interaction suggests that how closely the verb matched the walker affected reading times, as well. To investigate this interaction, we conducted pair-wise tests using linear mixed-effects models. These revealed that the interaction was driven by the Walker prime/Close-Match condition, which was read more slowly than both the Scrambled prime/Close-Match and Walker prime/Distant-Match conditions (*p*s < 0.05). As in Experiment 1, these results suggest that participants exhibited interference in processing closely-matching verbs after viewing a video of a point-light walker.

At region 3, there was only an interaction of Verb Match and Prime Type (β = −117.705, *SE* = 49.229, *t* = −2.391, *p* < 0.05), with no main effect of either Verb Match or Prime Type. To follow up on this interaction, pair-wise regressions were conducted and revealed that within the Close-Match verb condition, there was a marginal difference, such that Walker primes led to slower reading times than Scrambled primes (*p* = 0.07).

#### Literal sentences, static group

Mean reading times for Literal sentences for the Static group are plotted in Figure 4B. At region 1, there were no significant main effects of Verb Match or Prime Type, though there was a marginal interaction of Verb Match and Prime Type (β = −73.011, *SE* = 40.768, *t* = −1.791, *p* = 0.07).

At region 2, unlike in the Motion group, there were no main effects or interactions of any type.

At region 3, there was an interaction of Prime Type and Verb Match (β = −128.496, *SE* = 60.727, *t* = −2.116, *p* < 0.05). There was also a marginal effect of both Prime Type (β = 83.26, *SE* = 42.371, *t* = 1.965, *p* = 0.05) and Verb Match (β = 135.716, *SE* = 78.901, *t* = 1.72, *p* = 0.09). To tease apart the interaction of Prime Type and Verb Match, follow-up pair-wise regressions were performed. Within the Close-Match verb conditions, Walker primes led to slower reading times than Scrambled primes at region 3 (*p* < 0.05). Additionally, within the Scrambled prime conditions, there was a trend for the Distant-Match verb condition to lead to slower reading times than the Close-Match verb condition. Overall, the pattern of results at region 3 looks similar to the Motion group for Literal sentences, but the pattern at the preceding region (2) shows a different pattern (with similar reading times across conditions in the Static condition).

#### Metaphorical sentences, motion group

Mean reading times for Metaphorical sentences for the Motion group are plotted in Figure 3B. Unlike in Experiment 1, there were no significant differences at any of the three regions. However, at region 3, a numerical pattern similar to that seen in the Literal sentences, Motion group in Experiment 2 was observed, with the Distant-Match verb conditions leading to slower reading times compared to the Close-Match conditions.

#### Metaphorical sentences, static group

Mean reading times for Metaphorical sentences in the Static group are plotted in Figure 4B. At region 1, there was only a main effect of Prime Type, with Walker primes leading to longer reading times than Scrambled primes (β = 86.667, *SE* = 41.228, *t* = 2.102, *p* < 0.05).

At region 2, unlike in the Motion group, there were no main effects or interactions of any type.

At region 3, there was an interaction of Prime Type and Verb Match (β = 201.01, *SE* = 81.671, *t* = 2.461, *p* < 0.05). Follow-up tests showed that this interaction was driven by a difference within the Distant-Match verbs, with reading times at region 3 being slower after Walker primes than after Scrambled primes. These findings echo the results from the Metaphorical sentences in Experiment 1 (using video primes), where similar effects suggested interference following intact Walker primes for sentences containing Distant-Match verbs.

#### Comprehension questions

High accuracy was observed for comprehension questions in both the Motion group (*M* = 95.95%, *SD* = 5.85%) and the Static group (*M* = 97.14%, *SD* = 3.59%). For the Motion group, mean accuracy was 96.97% (*SD* = 4.13%) for filler sentences; 96.58% (*SD* = 5.24%) for literal sentences; and 95.29% (*SD* = 7.50%) for metaphorical sentences. For the Static group, mean accuracy was 97.74% (*SD* = 2.88%) for filler sentences; 97.42% (*SD* = 3.67%) for literal sentences; and 96.82% (*SD* = 5.29%) for metaphorical sentences. There were no differences based on Group, Sentence Type, Verb Match, or Prime Type (all *p*s > 0.10).

### Discussion: experiment 2

Experiment 2 used both moving and static primes. We found that conceptually, for Literal sentences, the findings from Experiment 1 were replicated: in the critical verb region, reading times were slowed for verbs closely matching the action depicted in the upright walker prime videos compared to other conditions. As in Experiment 1, these results suggest that participants exhibit interference in processing closely-matching verbs used literally after viewing a video of an upright point-light walker. However, for Metaphorical sentences, there were no main effects or interactions of Prime Type and Verb Match in the Motion group in Experiment 2 (unlike in Experiment 1), though a similar numerical pattern was observed for these sentences in the final region. The lack of replication may be due to an under-powered study, as we had slightly fewer participants per group in Experiment 2, and with perhaps not enough power to detect an interaction.

Literal sentences in the Static group followed a pattern similar to that of Literal sentences in the Motion group, though differences did not emerge until after the verb region; that is, they emerged at the final region (region 3). Here, an interaction of Verb Match and Prime Type emerged, with sentences containing Close-Match verbs leading to slower reading times after Walker primes compared to Scrambled primes. Interestingly, at region 3, sentences with Close-Match verbs were also read more slowly than sentences with Distant-Match verbs. This pattern is somewhat difficult to interpret, but taken together, these findings suggest a general difficulty for Close-Match verb sentences following Walker primes that emerged later in the sentence following still images than following video primes.

As for Metaphorical sentences in the Static group, the results suggest a similarity with Experiment 1 (where video primes were used), with an interaction of Prime Type and Verb Match at region 3 suggesting that sentences containing Distant-Match verbs are processed more slowly after Walker primes compared to Scrambled primes. However, as in the Literal sentences in the Static group, this interaction only emerged at region 3 whereas in Experiment 1, it emerged earlier (at the critical verb region, region 2). Although the pattern of results observed in Experiment 1 did not replicate in the Motion group of Experiment 2, still images, at least in this sample of participants, were able to differentially affect the processing times of sentences containing distantly- vs. closely-matching verbal content. These findings suggest that there may be partially overlapping neurocognitive representations for metaphorical language about biological motion and form-based visual content about biological motion.

## General discussion

In summary, we observed different patterns of reading times for metaphorical compared to literal sentences following biological motion video and (to some extent) still image primes. Across two experiments, for literal sentences, we observed a pattern that may roughly be connected with interference for sentences that contained verbs closely matching the sensorimotor content of the primes. However, in Experiment 1, for metaphorical sentences, we observed a pattern that suggested interference for sentences containing distantly-matching verbs. As for still images, we observed a similar pattern, except that effects were not observed at the verb region of sentences, but rather at a later prepositional phrase at the end of the sentence.

What type of mechanism could cause Close-Match verbs to be processed more slowly in literal sentences but more quickly in the metaphorical sentences? To the extent that an interference-type pattern emerged for the Close-Match verbs in the Literal condition following videos of point-light walkers, this pattern may have been due to difficulty in accessing a precise representation. For instance, if a participant had just read a Literal sentence with the phrase *was ambling*, she may have attempted to access a recently-activated representation of walking (recently accessed due to the viewing of a video prime of an upright point-light walker). However, if the recently-accessed representation did not constitute a close enough fit with the desired element in memory (e.g., a representation of *ambling*), the participant may have sensed an error due to a slight mismatch between the recently-accessed walking and the attempted access to *ambling*.

Relatedly, semantic priming paradigms have demonstrated that for *coordinates*, that is, categorically related words or pictures which might be used in many similar situations, participants often exhibit interference effects rather than facilitation in picture-naming tasks involving word primes (Alario et al., 2000; Sailor et al., 2009). Semantic priming studies have primarily been conducted using noun coordinates describing objects and animate beings, though one recent study in by Bergen et al. (2010) used pictures and words in a verification task to investigate actions and verbs. Participants were slower to reject mismatching picture-word pairs when the effector typically used to perform the action was the same across the picture and the word (e.g., a picture of someone running and the word *kick*). Here, the presence of interference for similar types of actions also suggests that people activate overlapping representations in visual and verbal modalities. In our study, the visual prime of a walking action and the verb region of the sentences similarly provide an analog to noun coordinate studies using action/verb coordinates. It is possible that for our literal sentences, residual activation of a walking action led to interference for processing the verb when it closely matched the walking action. For the Distant-Match verbs, it could be that participants were able to better use previously activated sensorimotor features from the walking action, with lack of precise match allowing for facilitation in a way that the closer-matching verbs did not achieve. This might be possible if it was easier to distinguish between the walking action and, e.g., verbs like *catapulting*, but both share some overall semantic features such as motion. This pattern is roughly analogous to findings from the semantic priming literature showing that semantic *associates* lead to facilitation under circumstances when semantic coordinates do not (Alario et al., 2000; Sailor et al., 2009). This is not to say that our videos (and static images) of a walking action and Distant-Match verbs like *catapulting* are in fact semantic associates, but a relationship remains between the two which is substantially different from the relationship between the walking action and our Close-Match verbs.

It is important to note that coordinate interference effects in picture-naming tasks are typically restricted to short priming latencies (Alario et al., 2000; Sailor et al., 2009). In our studies, latency for the presentation of the critical verb following the visual prime was variable, given that participants read each phrase at a self-paced rate, but on average, latencies were much longer (including the time between the prime and beginning to read the sentence as well as the time it took to read the first phrase of the sentence). To our knowledge, no other study has examined the timing dynamics of such priming effects of verbs in sentences.

It is difficult to know precisely why such differences for the Static group emerged later in the sentence. One possibility is that activation of sensorimotor semantic content following images may be slower-acting compared to videos. A related possibility is that videos increase the strength of activation levels (in a spreading-activation-type semantic network, e.g., Anderson, 1983). As for the Static primes, the information might not have been activated strongly enough to create such interference/facilitation effects until further down the line, when the content of the sentence had been processed more fully, possibly during so-called “sentence wrap-up” effects (Just and Carpenter, 1980; Rayner et al., 2000).

As for the Metaphorical sentences, it is unclear why the findings from Experiment 1 did not replicate in the Motion group in Experiment 2. It is possible that Experiment 2 was under-powered and that the numerical difference would be reliable in a larger group. To the extent that the findings from Experiment 1 are replicable, however, it appears that metaphorical language containing biological motion verbs may rely on less-specific sensorimotor representations compared to literal language, as indicated by the fact that interference-type effects were observed for the verbs which distantly, rather than closely, matched the content of the video primes. For the still images (Experiment 2), effects were again observed later in the sentence (as was the case for the Literal sentences), and may therefore reflect a similar phenomenon of either slower-acting or weaker activation of sensorimotor semantic content following images compared to videos.

Though our experimental sentences were matched for many lexical properties, the first noun of the metaphorical sentences was found to be more highly imageable for Close-Match conditions compared to Distant-Match conditions. Imageability is known to be highly correlated with concreteness, both of which are known to affect reading times (for critical word 1 in the experimental and filler items from the present study, imageability and concreteness were highly correlated, *r* = 88.55, *p* < 0.001). In general, high concreteness and imageability are thought to be associated with shorter reading times compared to lower concreteness and imageability (Holmes and Langford, 1976; Juhasz and Rayner, 2003), though this may be modulated by individual differences in a tendency to use imagery (Denis, 1982). However, our results indicated that for metaphorical sentences, differences in reading times at the verb region (just subsequent to processing the subject noun region) went in the opposite direction, with the more highly imageable Distant-Match condition leading to longer reading times during the verb region compared to the less highly imageable Close-Match condition. We argue that this pattern indicates a form of facilitation for more closely-matching verbs, which did not themselves differ in mean imageability across the Close- and Distant-match conditions. Given that the differences indicated by the *t*-test would bias our results in the opposite direction, we do not see this differences as terribly worrisome for our interpretation. However, future studies would do well to more carefully match this property across items.

The possibility that metaphors may be associated with the processing of less precise semantic content—that is, that they are associated with a broad distribution of content from semantic memory compared to literal language—is consistent with popular theories about right-hemisphere language processing. First, data from patients with right-hemisphere compared to left-hemisphere lesions (Winner and Gardner, 1977) and studies of healthy participants using methods from cognitive neuroscience (Mashal and Faust, 2008; Pobric et al., 2008) suggest preferential processing of metaphorical language by the right hemisphere, though not all experimental data support this interpretation (e.g., Coulson and Van Petten, 2007). Second, the right hemisphere may operate over less fine-grained linguistic representations than the left hemisphere. This “coarse coding” hypothesis (Beeman et al., 1994) suggests that there are hemispheric differences in specificity of coding of linguistic information, with the left hemisphere possibly honing in on specific words/concepts while the right hemisphere may activate a broader array of words and/or conceptual content. Our findings are consistent with the notion that broader arrays of semantic content may be activated during the processing of metaphorical language compared to literal language.

Mashal et al. (2007) suggest that the right hemisphere may not necessarily be specialized for metaphorical uses of language, *per se*, but rather for non-familiar language (similar to the “Graded Salience Hypothesis”; Giora, 1997). In an fMRI study, they observed an increased activation in the right hemisphere for novel metaphors (such as *pearl tears*), but not for conventional metaphors (such as *bright student*). Relatedly, the “career of metaphor” hypothesis (Bowdle and Gentner, 2005) proposes that as metaphors age, becoming more and more frequent in production, they become more crystallized so that the original, compositional meaning of their components is lost, and the full meaning of the metaphor is all that is retained. Under this hypothesis, proposals such as Lakoff's (1993) theory of conceptual metaphor are only relevant for new metaphors. That is, novel metaphors are more likely to be interpreted as mappings from a more concrete source domain to the more abstract target domain whereas older metaphors are more fixed in meaning (in the target domain). Recently, an fMRI study by Desai et al. (2011) found imaging evidence to support this hypothesis. They investigated sensorimotor metaphors which were rated as more or less familiar by an independent set of participants. An inverse correlation between familiarity of the metaphor and activation of primary motor areas was observed, such that more familiar metaphors activated these areas to a lesser extent. The authors took these findings as evidence for the (abstract) target of metaphors being “… understood in terms of the base domain through motoric simulations, which gradually become less detailed while still maintaining their roots in the base domain” (p. 10).

The metaphors used in the present studies are likely to be relatively new metaphors, by such definitions, which means that according to proposals like Bowdle and Gentner's (2005) “career of metaphor” theory and Mashal et al. (2007) hypothesis regarding the novelty of metaphors, these are the types of metaphors that are likely to engage the recruitment of sensorimotor representations in their processing. According to these theories, in doing so, they may also be likely to engage a different pattern of brain regions from those engaged when processing literal sentences. Our findings are consistent with a story wherein metaphorical language is processed in a different way from literal language, but still may recruit sensorimotor representations overlapping with the processing of visual biological motion and possibly implied motion (as suggested by the findings from the Static group in Experiment 2).

### Conflict of interest statement

The authors declare that the research was conducted in the absence of any commercial or financial relationships that could be construed as a potential conflict of interest.
