# Supplementary material for: Action verbs are processed differently in metaphorical and literal sentences depending on the semantic match of visual primes
Source: Front Hum Neurosci. 2014 Dec 4;8:982. doi: 10.3389/fnhum.2014.00982 (PMC4255517; doi:10.3389/fnhum.2014.00982)
Supplement: Supplementary file 1 [file Table1.DOCX]

Supplementary Materials

| **Critical Word** | **Sentence Type** | **Condition** | **Measure** | **Mean** | **(SD)** |
| --- | --- | --- | --- | --- | --- |
| Word 1 (Subject Noun) | Literal | Close | Concreteness | 542.86 | (46.06) |
|  |  | Distant |  | 582.80 | (32.39) |
|  |  | Close | Frequency | 52.50 | (45.90) |
|  |  | Distant |  | 78.56 | (110.20) |
|  |  | Close | Imageability | 564.86 | (52.65) |
|  |  | Distant |  | 594.00 | (44.11) |
|  |  | Close | Number of syllables | 2.22 | (0.44) |
|  |  | Distant |  | 1.78 | (1.09) |
|  | Metaphorical | Close | Concreteness | 426.14 | (52.59) |
|  |  | Distant |  | 526.25 | (104.58) |
|  |  | Close | Frequency | 117.13 | (98.92) |
|  |  | Distant |  | 95.38 | (100.82) |
|  |  | Close | Imageability | 441.14 | (58.37) |
|  |  | Distant |  | 560.00 | (53.00) |
|  |  | Close | Number of syllables | 2.38 | (0.74) |
|  |  | Distant |  | 2.00 | (0.93) |
| Word 2 (Verb) | Literal | Close | Concreteness | 393.67 | (67.32) |
|  |  | Distant |  | 473.50 | (65.03) |
|  |  | Close | Frequency | 25.80 | (41.86) |
|  |  | Distant |  | 24.00 | (45.75) |
|  |  | Close | Imageability | 457.67 | (49.64) |
|  |  | Distant |  | 542.33 | (37.71) |
|  |  | Close | Number of syllables | 2.56 | (0.73) |
|  |  | Distant |  | 2.44 | (0.88) |
|  | Metaphorical | Close | Concreteness | 393.67 | (67.32) |
|  |  | Distant |  | 471.80 | (72.56) |
|  |  | Close | Frequency | 31.25 | (46.24) |
|  |  | Distant |  | 25.50 | (49.93) |
|  |  | Close | Imageability | 457.67 | (49.64) |
|  |  | Distant |  | 536.40 | (38.90) |
|  |  | Close | Number of syllables | 2.63 | (0.74) |
|  |  | Distant |  | 2.50 | (0.93) |
| Word 3 (Final noun) | Literal | Close | Concreteness | 584.80 | (17.11) |
|  |  | Distant |  | 555.80 | (47.06) |
|  |  | Close | Frequency | 199.29 | (203.62) |
|  |  | Distant |  | 123.83 | (130.44) |
|  |  | Close | Imageability | 600.00 | (9.27) |
|  |  | Distant |  | 573.40 | (77.49) |
|  |  | Close | Number of syllables | 1.63 | (0.92) |
|  |  | Distant |  | 1.43 | (0.53) |
|  | Metaphorical | Close | Concreteness | 352.60 | (82.58) |
|  |  | Distant |  | 422.50 | (144.96) |
|  |  | Close | Frequency | 121.71 | (151.62) |
|  |  | Distant |  | 137.00 | (190.84) |
|  |  | Close | Imageability | 402.00 | (82.35) |
|  |  | Distant |  | 482.50 | (28.99) |
|  |  | Close | Number of syllables | 2.29 | (1.11) |
|  |  | Distant |  | 1.80 | (0.84) |

Supplementary Table 1. Mean and standard deviation (SD) for close- and distant-match conditions for each critical word and sentence type for concreteness, frequency, imageability, and number of syllables. Measures were obtained from the MRC Psycholinguistic Database (Wilson, 1988). Concreteness and imageability are derived from norms taken from Coltheart (1981), and frequency is based on the Kucera-Francis (1967) written frequencies.

References

Coltheart, M. (1981). MRC Psycholinguistic Database User Manual: Version 1. Available online: http://www.psych.rl.ac.uk/User_Manual_v1_0.html

Kucera, H & Francis, W.N. (1967). Computational Analysis of Present-Day American English. Providence: Brown University Press.
