# Supplementary material for: Action verbs are processed differently in metaphorical and literal sentences depending on the semantic match of visual primes
Source: Front Hum Neurosci. 2014 Dec 4;8:982. doi: 10.3389/fnhum.2014.00982 (PMC4255517; doi:10.3389/fnhum.2014.00982)
Supplement: Supplementary file 2 [file Table2.DOCX]

Supplementary materials

| **Critical Word** | **Sentence Type** | **Measure** | **t-statistic** | **p-value** |
| --- | --- | --- | --- | --- |
| Word 1 (Subject Noun) | Literal | Concreteness | -1.76 | 0.11 |
|  |  | Frequency | -0.65 | 0.53 |
|  |  | Imageability | -1.09 | 0.30 |
|  |  | Num. Syllables | 1.13 | 0.28 |
|  | Metaphorical | Concreteness | -1.79 | 0.15 |
|  |  | Frequency | 0.44 | 0.67 |
|  |  | Imageability | -3.45 | 0.01 |
|  |  | Num. Syllables | 0.89 | 0.39 |
| Word 2 (Verb) | Literal | Concreteness | -1.70 | 0.17 |
|  |  | Frequency | 0.07 | 0.95 |
|  |  | Imageability | -2.60 | 0.07 |
|  |  | Num. Syllables | 0.29 | 0.77 |
|  | Metaphorical | Concreteness | -1.54 | 0.19 |
|  |  | Frequency | 0.19 | 0.86 |
|  |  | Imageability | -2.35 | 0.09 |
|  |  | Num. Syllables | 0.30 | 0.77 |
| Word 3 (Final noun) | Literal | Concreteness | 1.30 | 0.25 |
|  |  | Frequency | 0.81 | 0.44 |
|  |  | Imageability | 0.76 | 0.49 |
|  |  | Num. Syllables | 0.51 | 0.62 |
|  | Metaphorical | Concreteness | -0.64 | 0.62 |
|  |  | Frequency | -0.14 | 0.90 |
|  |  | Imageability | -1.91 | 0.11 |
|  |  | Num. Syllables | 0.86 | 0.41 |

Supplementary Table 2. T-test results for close- vs. distant-match conditions for each critical word and sentence type for concreteness, frequency, imageability, and number of syllables. See Supp. Table 1 caption for descriptions of these variables.
